# Utility of bronchoscopically obtained frozen cytology pellets for next-generation sequencing

**DOI:** 10.1186/s12885-024-12250-5

**Published:** 2024-04-17

**Authors:** Chihiro Mimura, Rei Takamiya, Shodai Fujimoto, Takafumi Fukui, Atsuhiko Yatani, Jun Yamada, Mizuki Takayasu, Naoya Takata, Hiroki Sato, Kiyoko Fukuda, Koichi Furukawa, Daisuke Hazama, Naoko Katsurada, Masatsugu Yamamoto, Shingo Matsumoto, Koichi Goto, Motoko Tachihara

**Affiliations:** 1 Division of Respiratory Medicine, Department of Internal Medicine, Kobe University Graduate School of Medicine, 7-5-1 Kusunoki-Cho, Chuo-Ku, Kobe-City, Hyogo, 650-0017 Japan; 2 Department of Thoracic Oncology, National Cancer Center Hospital East, 6-5-1 Kashiwanoha, Kashiwa-City, Chiba, 277-8577 Japan

**Keywords:** Lung cancer, Next-generation sequencing (NGS), Pellet specimen, Cytology, Endobronchial ultrasound-guided transbronchial needle aspiration (EBUS-TBNA)

## Abstract

**Background:**

Next-generation sequencing (NGS) is essential for lung cancer treatment. It is important to collect sufficient tissue specimens, but sometimes we cannot obtain large enough samples for NGS analysis. We investigated the yield of NGS analysis by frozen cytology pellets using an Oncomine Comprehensive Assay or Oncomine Precision Assay.

**Methods:**

We retrospectively enrolled patients with lung cancer who underwent bronchoscopy at Kobe University Hospital and were enrolled in the Lung Cancer Genomic Screening Project for Individualized Medicine. We investigated the amount of extracted DNA and RNA and determined the NGS success rates. We also compared the amount of DNA and RNA by bronchoscopy methods. To create the frozen cytology pellets, we first effectively collected the cells and then quickly centrifuged and cryopreserved them.

**Results:**

A total of 132 patients were enrolled in this study between May 2016 and December 2022; of them, 75 were subjected to frozen cytology pellet examinations and 57 were subjected to frozen tissue examinations. The amount of DNA and RNA obtained by frozen cytology pellets was nearly equivalent to frozen tissues. Frozen cytology pellets collected by endobronchial ultrasound-guided transbronchial needle aspiration yielded significantly more DNA than those collected by transbronchial biopsy methods. (*P* < 0.01) In RNA content, cytology pellets were not inferior to frozen tissue. The success rate of NGS analysis with frozen cytology pellet specimens was comparable to the success rate of NGS analysis with frozen tissue specimens.

**Conclusions:**

Our study showed that frozen cytology pellets may have equivalent diagnostic value to frozen tissue for NGS analyses. Bronchial cytology specimens are usually used only for cytology, but NGS analysis is possible if enough cells are collected to create pellet specimens. In particular, the frozen cytology pellets obtained by endobronchial ultrasound-guided transbronchial needle aspiration yielded sufficient amounts of DNA.

**Trial registration:**

This was registered with the University Medical Hospital Information Network in Japan (UMINCTR registration no. UMIN000052050).

**Supplementary Information:**

The online version contains supplementary material available at 10.1186/s12885-024-12250-5.

## Introduction

In lung cancer treatment, it is essential to investigate targetable driver mutations to evaluate indications for molecular-targeted therapy [1–3]. In patients with targetable driver gene mutations such as epidermal growth factor receptor (*EGFR*), fusion of echinoderm microtubule-associated protein-like 4 (*EML4*) and anaplastic lymphoma kinase (*ALK*), ROS proto-oncogene 1 (*ROS1*), v-raf murine sarcoma viral oncogene homolog B1 (*BRAF) V600E*, and mesenchymal-epithelial transition factor (*MET)* exon 14 skipping mutations, molecularly targeted therapy boasts longer progression-free survival than cytotoxic chemotherapy [4–8]. For this reason, it is very important to detect treatable driver mutations. In the past, single-driver mutation tests were mainly used, whereas in recent years, next-generation sequencing (NGS) has been used more frequently, as it can simultaneously investigate multiple genes [9, 10].

Suitability for a multi-gene panel test analysis was evaluated based on cell count and tumor tissue rate [11]. It is usually performed on formalin-fixed paraffin-embedded (FFPE) samples. For multi-gene panel tests, the tumor cell content must be at least 20–30% [12]. The recommended FFPE slide size is 5 µm × 10, and the amount of DNA needed for a panel test is 10–500 ng [13]. However, sometimes only minute tissue specimens or liquid specimens can be obtained due to lesion site or size and/or patient condition. If the defined tumor cell count requirements are not met, a false negative result may be obtained [10]. FFPE also reportedly has a lower NGS success rate than frozen specimens [14].

The lung cancer compact panel (LCCP), which was recently approved as a new multi-companion diagnostic method, uses cytology pellet specimens and has very high sensitivity for detecting genetic mutations [15]. Another report showed that cytology pellet specimens from the fixative medium of transbronchial lung biopsy can exfoliate tumor cells with good cellularity [16]. It was previously reported that, although the amount of DNA and RNA is lower than those with other methods, transbronchial brushing cytology pellet specimens did not have inferior NGS success rates to tissue specimens [17]. Although there are reports of the usefulness of cytology for the pathological diagnosis of lung cancer [18, 19], it is important to assess whether pellet samples are useful for NGS.

Our institution participated in the Lung Cancer Genomic Screening Project for Individualized Medicine in Asia (LC-SCRUM-Asia) (UMIN ID: UMIN000010234), or in the former LC-SCRUM (UMIN ID: UMIN000036871), a prospective nationwide genomic screening study for lung cancer [20, 21]. LC-SCRUM-Asia involves more than 200 medical centres across Japan. The study was designed to simultaneously examine multiple genetic alterations, including rare genetic alterations in cancer. Here we aimed to determine the DNA and RNA concentrations and cancer-related genes of the submitted samples in cases enrolled in the LC-SCRUM-Asia and investigated the usefulness of NGS for examining frozen cytology pellet specimens. Moreover, we examined the amount of DNA and RNA extracted from the pellets versus tissue specimens.

## Methods

### Study design and patients

We retrospectively enrolled patients who underwent bronchoscopy at Kobe University Hospital between May 2016 and December 2022 in the LC-SCRUM-Asia or the former LC-SCRUM. Case enrollment in LC-SCRUM-Asia would begin on June 1, 2019, and the former LC-SCRUM would begin on March 13, 2013. The inclusion criteria of this study were age ≥ 16 years, stage II–IV or recurrence of lung cancer, Eastern Cooperative Oncology Group (ECOG) performance status score of 0–1, preserved organ function, and no serious comorbidities. We examined the amount of extracted DNA and RNA and the success rate of the genetic mutation analysis using an Oncomine Comprehensive Assay ver. 1 or 3 (March 2015 to January 2021) or an Oncomine Precision Assay (January 2021 to December 2022). Patients who were asked not to participate in this study based on publicly available information were excluded.

We collected information such as age, sex, smoking history, primary tumor size, histology, and clinical stage based on the 8^th^ edition of the TNM classification, bronchoscopy procedure, and sample data collected from the patients’ medical records.

Informed consent was obtained from all the enrolled patients. This study was approved by the Kobe University Ethics Committee (230046) on July 19, 2023. This study was conducted in accordance with the principles of the Declaration of Helsinki. This was registered with the University Medical Hospital Information Network in Japan (UMINCTR registration no. UMIN000052050).

### Bronchoscopy methods

All bronchoscopes and devices were manufactured by Olympus, Tokyo, Japan. Bronchoscopy procedures included endobronchial biopsy (EBB), transbronchial biopsy (TBB) under X-ray fluoroscopy, TBB (ultrathin), endobronchial ultrasound with a guide sheath transbronchial biopsy (EBUS-GS-TBB), and endobronchial ultrasound-guided transbronchial needle aspiration (EBUS-TBNA). For the TBB under X-ray fluoroscopy, we did not use a guide sheath and performed a biopsy while confirming the lesion on radiography. In EBUS-GS-TBB methods, guide sheaths were available in thick and thin diameters. Differences were noted between the working channel and forceps diameter in the thin and thick arms. We used the thick bronchoscope 1T260 (5.9-mm outer diameter, 2.8-mm working channel diameter) or 1TQ290 (5.9-mm outer diameter, 3.0-mm working channel diameter), and the thin bronchoscope P290 (4.2-mm outer diameter, 2.0-mm working channel diameter) or P260F (4.0-mm outer diameter, 2.0-mm working channel diameter). The forceps diameter was 1.9 mm in the thick bronchoscope arm and 1.5 mm in the thin bronchoscope arm. An ultrafine fiber without a guide sheath was used for the ultrathin bronchoscopy. We used an ultrathin bronchoscope (MP290F; 3.0-mm bronchoscope diameter, 1.7-mm working channel diameter). For EBUS-TBNA, we used a UC260FW or UC290 bronchoscope with 22-gauge needles.

We discuss which lesion was suitable for pathology before bronchoscopy in all cases, such as bronchoscope type and lesion location. We used TBB method if the lesion was in the lungs, while if the lesion was in both lungs and lymph nodes, the decision whether we use EBUS-TBNA or TBB method was based on CT scan images, including the presence of necrosis, location of blood vessels and bronchoscopic findings. We focused on selecting lesions where more specimens could be obtained and NGS analysis was considered feasible.

### Specimens collection

We diagnosed primary lung cancer by Papanicolaou and Hematoxylin–Eosin staining of specimens collected by bronchoscopy. In cases where sufficient tumor cells were obtained, frozen specimens were submitted to LC-SCRUM. It was decided that only tissue or pellet specimens should be submitted as frozen specimens in LC-SCRUM. Because LC-SCRUM provided data for clinical research purposes, NGS analysis using Oncomine Dx Target Test (ODxTT) by FFPE was also performed at the same time in cases with sufficient tumor cells to be used as data in clinical practice.

**Fig. 1:**
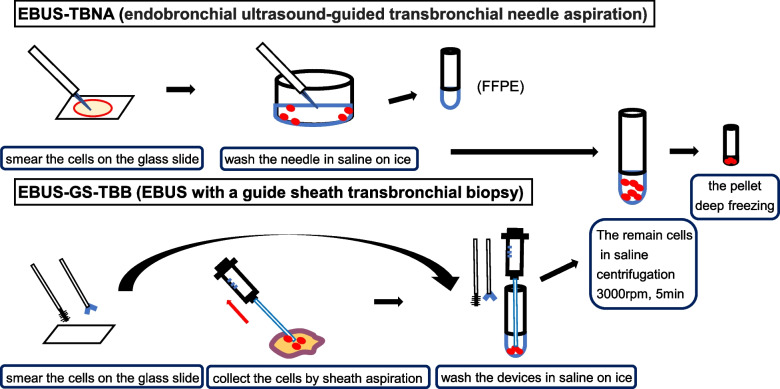
How to make the pellets. Legends: Schema of the procedure used to make the frozen cytology pellets. First, we smeared the cells on the glass slide. Next, we washed the devices in saline on ice. We collected the remaining cells in saline, and cryopreserved them as cytology pellets

For analysis in LC-SCRUM, in frozen tissue arm, 1 or 2 of the tissue specimens obtained were cryopreserved. In frozen cytology pellet arm, we first smeared cells on the glass slide of the EBUS-TBNA arm and the TBB arm. In EBUS-TBNA arm, one drop of cytology specimens by needle stylet was smeared on the slide glass after puncture. Next, tissue specimens were pushed out with air and saline on ice. After pushing out histologic core, we removed tissue specimens from saline and used them for FFPE. The remain cells in saline was stored as a pellet. In TBB method, we performed forceps biopsy in all cases. In almost cases, we performed bronchial brushing. We smeared cell on the glass slides at first and we collected cytological specimens by washing devices in saline on ice. We used the guide sheath aspiration method in EBUS-GS-TBB methods. After forceps biopsy and bronchial brushing, we moved the guide sheath back and forth with 20 mL of negative pressure suction for 20 s to collect the cytology specimen contained within sheath lavage fluid. This is because we found that the aspiration method had a higher diagnostic rate in cell block specimens than the usual method which we only washed guide sheath without suction after biopsy [22]. After we collected the cell specimens, they were centrifuged as soon as possible, and only the pellet was deep-frozen at -80 °C (Fig. 1).

### Extracted DNA and RNA and NGS analysis

All frozen specimens were carried to the LSI Medience Corporation. DNA and RNA extractions were performed using a nucleic acid extraction kit (Alleprep DNA/RNA Mini Kit; QIAGEN). DNA and RNA concentrations were determined using a Qubit fluorometric assay (Thermo Fisher Scientific).

The NGS analysis was performed using a hot-spot cancer panel (Oncomine Comprehensive Assay ver. 1.0 or 3.0 or Oncomine Precision Assay) [23, 24], which identified 161 and 50 cancer-related genes, respectively. We also identified 20 treatable gene mutations: *EGFR* mutations, human epidermal growth factor receptor 2 (*HER2)* mutations, KRAS proto-oncogene (*KRAS)* mutations, neuroblastoma RAS viral (v-ras) oncogene homolog (*NRAS)* mutations, Harvey rat sarcoma viral oncogene homolog (*HRAS)* mutations, *BRAF* mutations, *MET* mutations, AKT serine *1* mutations, phosphatidylinositol-4,5-bisphosphate 3-kinase catalytic subunit alpha (*PIK3CA*) mutations, fibroblast growth factor receptor (FGFR) *1–4* mutations, Rearranged during transfection (*RET)* fusion genes, *ALK* fusion genes, *ROS1* fusion genes, neurotrophic receptor tyrosine kinase (*NTRK)1–3* fusion genes, and Neuregulin-1 (*NRG1)* fusion genes.

An NGS analysis requires the submission of at least 10 ng of DNA or RNA. The minimum concentration of DNA was defined as 1.67 ng/μL and that of RNA as 2.5 ng/μL. If the concentration of DNA or RNA is insufficient or the nucleic acid quality is compromised, an NGS analysis cannot be performed. Data of the amount of extracted DNA and RNA and NGS analyses were reported to our hospital. We also performed an NGS analysis with FFPE using the ODxTT in some cases.

### Statistical analysis

Patients characteristics and DNA and RNA concentrations of the frozen tissue versus frozen pellet groups were determined using the Mann–Whitney *U* test. Patients characteristics and the NGS success rate for each group was determined using Fisher’s exact test. DNA and RNA concentrations were determined by bronchoscopy methods using the Kruskal–Wallis test. All statistical analyses were performed using EZR version 1.55 (Saitama Medical Center, Jichi Medical University, Saitama, Japan), a graphical user interface for R (ver. 3.6.3; The R Foundation for Statistical Computing, Vienna, Austria) [25]. Values of *p* < 0.05 were considered statistically significant.

## Results

### Patient characteristics

From May 2016 to December 2022, 132 patients were enrolled. The patient characteristics are shown in Table 1. Frozen cytology pellets were examined in 75 cases versus tissue specimens in 57 cases. The median patient age was 71 (range, 39–86) years in the frozen cytology pellet arm and 72 (range, 35–85) years in the frozen tissue arm. Males accounted for 77.3% (*n* = 58) of the frozen cytology pellet arm and 75.4% (*n* = 43) of the frozen tissue arm. By histologic types, non-squamous non-small cell lung cancer (NSCLC) types such as adenocarcinoma and large cell carcinoma accounted for 65.4% (*n* = 49) of the frozen cytology pellet arm versus 54.4% (*n* = 31) of the frozen tissue arm. There were no significant differences in age, sex, Brinkman index, clinical stage, or histology between the frozen cytology pellet and the frozen tissue arms. The median primary tumor size was larger in the frozen tissue versus frozen cytology pellet arm (*p* < 0.01). EBUS-TBNA was performed more frequently in the frozen cytology pellet arm since we attempted to submit more frozen pellet specimens for it. The number of biopsies taken by the TBB methods has shown in Supplementary Table 1. Bronchial brushing was performed one or two times.

**Table 1:** Patients characteristics

	Frozen Cytology Pellet (*n* = 75)	Frozen Tissue (*n* = 57)	ORR (95%CI)	*P* value
Age-Median [IQR]	72 [65.5–76]	71 [66–74]		0.47
male/female	58/17	43/14	0.9 (0.37–2.21)	0.84
Brinkman Index Median [IQR]	900 [440–1210]	940 [570–1060]		0.69
primary tumor size(mm) Median [IQR]	35 [25–46]	50 [35–63]		< 0.01
Stage				0.08
II	3 (4.0%)	9 (15.8%)		
III	32 (42.7%)	17 (29.8%)		
IV	38 (50.6%)	29 (50.9%)		
Recurrent	2 (2.7%)	2 (3.5%)		
Histology				0.37
Adeno	39 (52.0%)	27 (47.4%)		
Sq	17 (22.6%)	21 (36.8%)		
NOS	9 (12.0%)	4 (7.0%)		
SCLC	9 (12.0%)	5 (8.8%)		
LCNEC	1 (1.4%)	0		
Procedure				< 0.01
EBUS-TBNA	31 (41.3%)	3 (5.3%)		
TBB (ultrathin)	1 (1.4%)	1 (1.7%)		
EBUS-GS-TBB (thin)	27 (36.0%)	21 (36.8%)		
EBUS-GS-TBB (thick)	4 (5.3%)	11 (19.3%)		
EBB	10 (13.3%)	18 (31.6%)		
TBB under X-ray fluoroscopy	2 (2.7%)	3 (5.3%)		

*Adeno* adenocarcinoma, *Sq* squamous cell carcinoma, *NOS* not other specified, *SCLC* small cell carcinoma, *LCNEC* large cell neuroendocrine carcinoma, *EBUS-TBNA* endobronchial ultrasound-guided transbronchial needle aspiration, *TBB* transbronchial biopsy, *EBUS-GS-TBB* endobronchial ultrasound with a guide sheath transbronchial biopsy, *EBB* endobronchial biopsy

### Amounts of extracted DNA and RNA of frozen cytology pellets versus tissues

Table 2 shows the amounts of extracted DNA and RNA. In the frozen cytology pellet arm, the median extracted yield of DNA was 60 (range, 3–340) ng/mL, while that of RNA was 10 (range, 2–260) ng/mL. In the frozen tissue arm, the median extracted yield of DNA was 40 (range 1–280) ng/mL, while that of RNA was 10 (range, 2–220) ng/mL. The frozen cytology pellets and frozen tissues had similar amounts of DNA and RNA.

**Table 2:** Amount of extracted DNA and RNA, NGS success rate

	Frozen Cytology Pellet (*n* = 75)	Frozen Tissue (*n* = 57)	ORR (95%CI)	*P* value
Extracted amount (ng/mL)Median [IQR]				
DNA	60 [2–100]	40 [3–70]		0.34
RNA	10 [9.5–30]	10 [5–29]		0.4
NGS success rate (%)				
DNA sequencing	97.3%	98.2%	1.5 (0.08–90.45)	0.99
RNA sequencing	76.0%	77.2%	1.16 (0.47–2.93)	0.99
	Frozen Cytology Pellet (*n* = 31)	Frozen Tissue (*n* = 3)		*P* value
EBUS-TBNA NGS success rate (%)				
DNA sequencing	100.0%	100.0%		
RNA sequencing	83.9%	33.3%	0.11 (0.002–2.4)	0.1

**Fig. 2:**
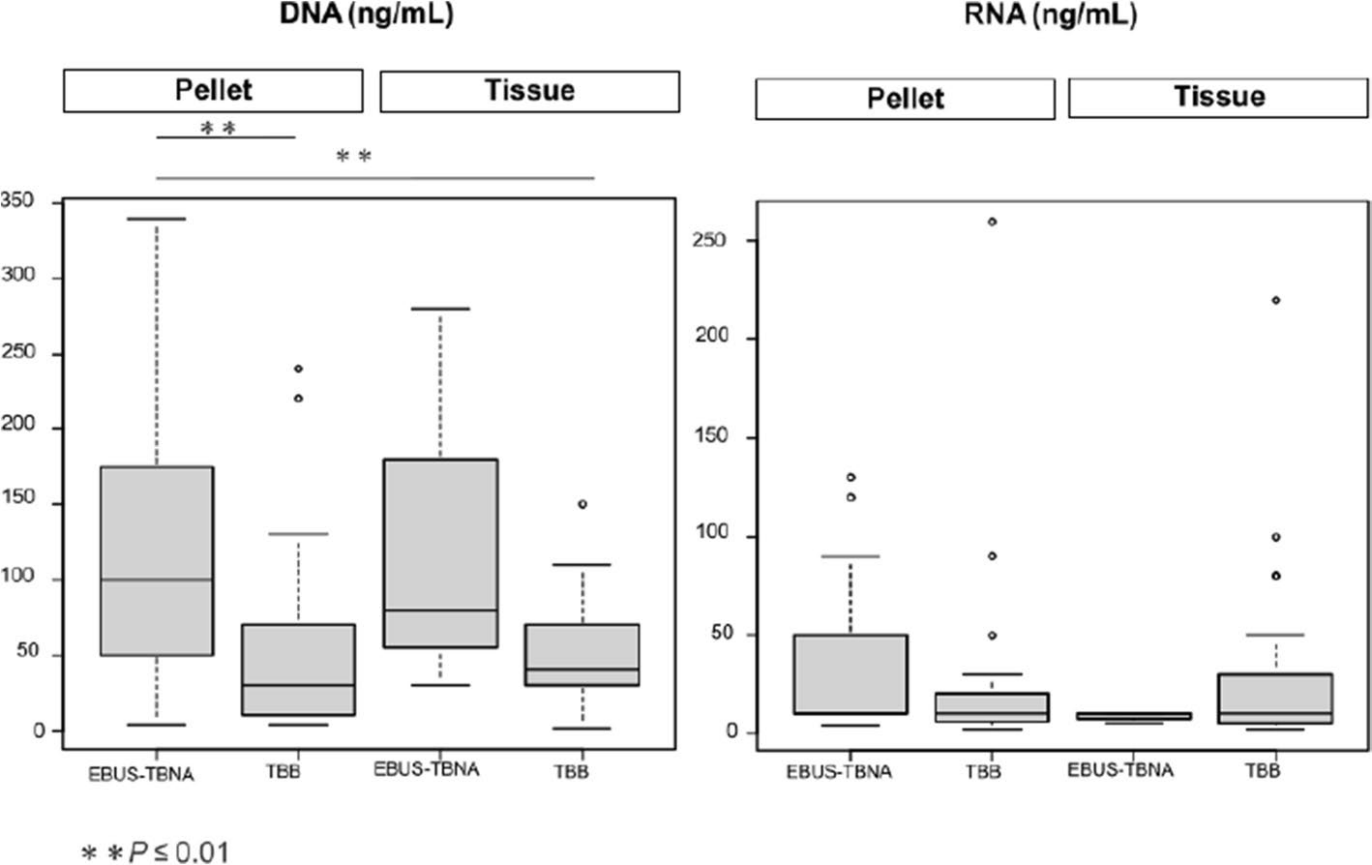
Extracted DNA and RNA yielded according to the procedure. Legends: TBB methods were further divided into TBB (ultrathin), EBUS-GS-TBB (thin), EBUS-GS-TBB (thick), EBB, and TBB under X-ray fluoroscopy. EBB, endobronchial biopsy; EBUS-GS-TBB, endobronchial ultrasound with a guide sheath transbronchial biopsy; TBB, transbronchial biopsy

The frozen cytology pellets obtained by EBUS-TBNA yielded significantly more DNA than those obtained by the TBB method (*p* < 0.01) (Fig. 2). There were no significant differences in the amount of extracted RNA in the respective frozen cytology pellets and frozen tissues between the EBUS-TBNA and TBB methods. TBB methods were further divided into TBB (ultrathin), EBUS-GS-TBB (thin), EBUS-GS-TBB (thick), EBB, and TBB under X-ray fluoroscopy. The frozen cytology pellets obtained by EBUS-TBNA yielded significantly more DNA than both the frozen cytology pellet and tissues obtained by EBUS-GS-TBB (thin). The frozen cytology pellets obtained by EBUS-TBNA yielded significantly more RNA than the frozen tissues obtained by EBUS-GS-TBB (thin) (*p* < 0.05). RNA was collected equally well in both pellet and tissue groups by other TBB methods (Supplementary Fig. 1).

### Analysis of NGS success rate

The NGS success rate for DNA was 97.3% in the frozen cytology pellet arm and 98.2% in the frozen tissue arm. The NGS success rate for RNA was 76.0% in the frozen cytology pellet group and 77.2% in the frozen tissue group (Table 2). There were no significant differences in NGS success rates for DNA or RNA between the frozen cytology pellets and the frozen tissues.

Particularly in EBUS-TBNA, the DNA extraction success rate was 100.0% for the frozen cytology pellets and frozen tissues, whereas the RNA extraction success rate was 83.9% in the frozen cytology pellet arm versus 33.3% in the frozen tissue arm. Thus, the success rate of the RNA extraction tended to be higher in the frozen cytology pellets than in the frozen tissues.

In the frozen cytology pellet arm, we examined the clinical factors related to NGS success rate but found no significant differences (Table 3). Particularly in EBUS-TBNA, we examined a number of punctures and bronchoscopes but found no significant differences among them.

**Table 3:** Analysis of clinical factors for NGS success rate in cytology pellet

		Univariate	
		ORR (95%CI)	*P*-value
**Factor**	***n*= 75**		
Age			
≥ 75	28 (37.3%)	1	
< 74	47 (62.7%)	2.516 (0.676–11.82)	0.167
Sex			
Male	58 (77.3%)	1	
Female	17 (22.8%)	1.435 (0.333–11.54)	0.536
Histology			
Adeno	39 (52.0%)	1	
Non-adeno	36 (48.0%)	0.618 (0.176–2.043)	0.427
Procedure			
EBUS-TBNA	31 (41.3%)	1	
TBB	44 (58.7%)	2.159 (0.618–8.791)	0.272
**Pellet specimens by EBUS-TBNA**	***n* = 31**		
Bronchoscope			
UC290	15 (48.4%)	1	
UC260FW	16 (51.6%)	1.481 (0.144–20.52)	1
A number of puncture			
≥ 4	8 (25.8%)	1	
< 3	23 (74.2%)	0.463 (0.042–6.761)	0.583

### Concordance of NGS analysis by frozen cytology pellets and FFPE

EBUS-TBNA tended to have a higher RNA extraction success rate than TBB. We examined the NGS analysis concordance rate by frozen cytology pellets and FFPE in EBUS-TBNA. Twenty-two of the 132 cases performed both ODxTT by FFPE and NGS analysis by Oncomine Comprehensive Assay ver. 1.0 or 3.0 or Oncomine Precision Assay on frozen cytology pellet specimens in LC-SCRUM (Supplementary Table 2). Of these, 15 cases (68%) were successfully analyzed using both FFPE and frozen cytology pellets. Six cases (27%) were successfully analyzed only by FFPE frozen specimens, but 4 of these cases were by TBB methods. In one EBUS-TBNA case, NGS analysis by FFPE was difficult due to poor RNA quality, but NGS analysis by frozen cytology pellets was successful.

### Genetic alterations

**Fig. 3:**
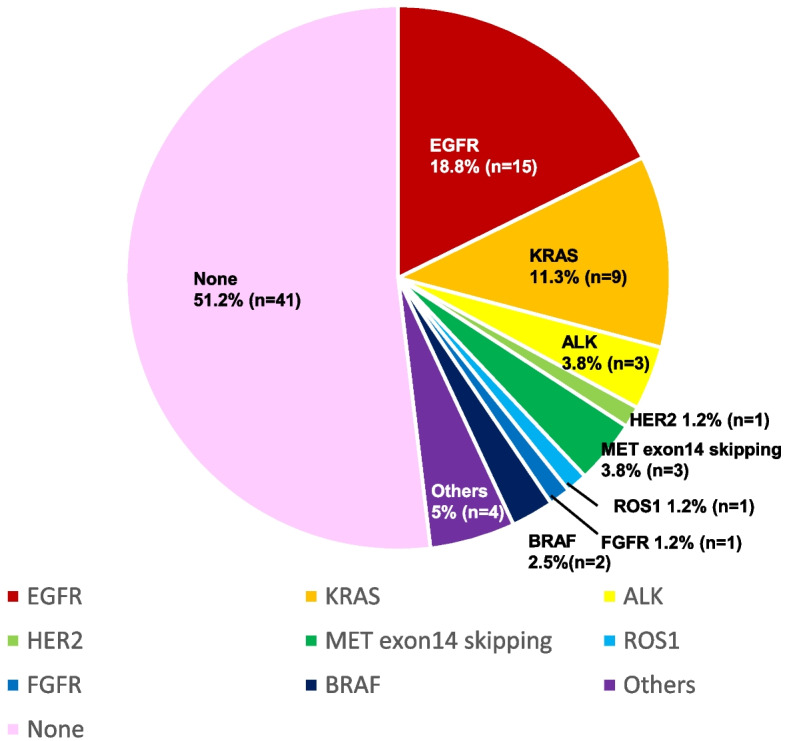
Genetic alterations obtained by NGS analysis. Legends: The percentages and the number of patients of targetable genetic alterations in non-squamous non-small cell lung cancer (*N* = 80). Others included two driver mutations: GNAS and PIKC3A

In non-squamous NSCLC, we detected genetic alterations in 48.8% of cases (Fig. 3). According to *EGFR* mutation type, 15 patients (18.8%) had an *EGFR* mutation, five (6.3%) harbored an exon 19 deletion, eight (10%) harbored a point mutation in exon 21 resulting in an L858R substitution, and two (2.5%) harbored other mutations. The details of other genetic alterations were as follows: *KRAS G12C* mutation (*n* = 2 [2.5%]), *KRAS* other mutation (*n* = 7, 8.8%), *EML-ALK* fusion (*n* = 3 [3.8%]), *HER2* exon 20 insertion (*n* = 1 [1.2%]), *MET* exon 14 skipping mutation (*n* = 3 [3.8%]), *ROS1* fusion (*n* = 1 [1.2%]), FGFR (*n* = 1 [1.2%]), *BRAF V600E* mutation (*n* = 1 [1.2%]), and *BRAF* other mutation (*n* = 1 [1.2%]). Others included two driver mutations: GNAS complex locus (*GNAS)* and *PIKC3)*.

## Discussion

This was the first study to investigate the usefulness of frozen cytology pellets for NGS analysis, particularly by using a bronchoscopy procedure. Specimens yielded by device washing are commonly used for cytology. However, via the collection of a large number of cells and creating cytology pellet specimens, it was possible to perform NGS analysis on these specimens. We found that frozen cytology pellet specimens had similar amounts of extracted DNA and RNA as tissue specimens. Frozen cytology pellets obtained by EBUS-TBNA yielded significantly more DNA than those obtained by TBB methods. In TBB methods classification, both cytology pellets and frozen tissue specimens obtained by EBUS-GS-TBB (thin) yielded significantly less DNA than cytology pellet specimens obtained by EBUS-TBNA. One reason for this may be that the specimens obtained with the small bronchoscope were significantly smaller than those obtained with the large bronchoscope [26]. EBUS-TBNA may be superior to TBB for obtaining frozen tissue samples from central lesions [27], while the bronchoscopy procedure should also be selected according to lesion site and size. In cases where the primary lesion is necrotic or where EBUS findings suggest that the probe is adjacent to the lesion but does not reach the interior of the lesion, we are likely to be unable to obtain an adequate specimen by TBB methods. In such cases, EBUS-TBNA should be selected if there are lymph nodes that can be punctured.

On the other hand, the RNA extraction success rate from the frozen tissue obtained by EBUS-TBNA was very low. These results differed from those reported using polymerase chain reaction–based tissue specimens [28]. NGS analysis using frozen tissue obtained by EBUS-TBNA was unsuccessful in two of the three cases. These two cases had more than three punctures. In both cases, NGS analysis using FFPE was not performed, but histology type could be diagnosed by FFPE. It is possible that the quantity of samples submitted as frozen specimens was small or that the freezing took longer due to poor RNA quality. The small number of cases may also have been a contributing factor.

We also found that the NGS success rate was comparable between frozen cytology pellets and frozen tissue. This result was similar to the previously reported NGS success rates of pellet specimens [17]. NGS analyses are most often performed on FFPE. This was probably because tissue specimens provide information about tumor cell counts. On the other hand, fresh-frozen specimens are used in the LC-SCRUM-Asia. As formalin fixation is not performed, nucleic acid quality is more likely preserved in frozen specimens.

Our study showed a dissociation between DNA and RNA concentrations. A previous report showed that the prolonged storage of FFPE reduces the detection rate of gene mutations [29]. It is also possible that the RNA in FFPE is degraded [30]. We believe that processing specimens on ice and freezing them quickly may maintain nucleic acid quality and enable appropriate gene mutation searches. To increase the amount of extracted RNA, the use of an RNase inhibitor might be useful [15]. Preserving RNA quality, especially when unstable, is critical for NGS analysis success.

Our study found no significant differences among clinical factors and bronchoscopy procedures in terms of NGS success rate. A diagnosis was reportedly possible using three punctures, but a minimum of four punctures was desirable for NGS [31]. It is advisable to ensure the number of punctures and biopsies and collect the necessary specimen volume for the NGS analysis.

There were cases in which the NGS analysis was possible with FFPE, but not with frozen cytology pellet specimens. This may be because the cytology pellets could not be confirmed to contain a sufficient number of tumor cells. It is important to confirm tumor cell counts in pellet specimens by creating the cell blocks whenever possible. Further advantages of making cell blocks include the ability to determine tissue architecture more easily than cytology pellets and the ability to make a diagnosis by immunohistochemical staining. However, the making cell blocks halves the number of specimens that can be used as frozen pellets. Cell blocks are also susceptible to RNA damage by formalin. In cases in which NGS could not be performed using frozen cytology pellets, the relevance of features such as tumor necrosis and differentiation are unknown. Two of our cases were not analyzed. Previous reports suggested that fragile tumor tissues may include fewer tumor cells [16], we believe that this may be due to high tumor differentiation; moreover, cell binding was strong and the pellet may not contain tumor cells. An LCCP was recently used, and with the help of rapid on-site cytologic evaluation (ROSE) [32], it would be possible to perform genetic mutation testing on pellet samples on the day of testing. The usefulness of NGS analysis by cryobiopsy has also been reported [33]. Although we did not obtain data on whether or not ROSE was performed in this study, we are now using ROSE to enable more reliable genetic analysis. We must continue to devise various ways to improve NGS analysis accuracy.

This study has several limitations. First, it was a single-center cohort because it used only the LC-SCRUM-Asia data. Second, this study had a small sample size, so only three tissue specimens were obtained by EBUS-TBNA. We also could not identify clinical factors related to NGS success rate. Third, we had no data on tumor cell counts on FFPE in some cases since such data would not have been measured when bronchoscopy was performed long ago. In cases in which an NGS analysis could not be performed, we plan to investigate tumor cell counts and pathological characteristics in the future. Finally, we were unable to compare tissue and cytology pellet NGS analyses of the same patients. When we submit specimens to LC-SCRUM, we need to choose frozen tissue or cytology pellets for NGS analysis. Therefore, it was difficult to investigate both frozen specimens simultaneously. Further case data is needed.

## Conclusions

In NGS, bronchoscopy frozen cytology pellets may have equivalent diagnostic value as frozen tissue. Frozen cytology pellets obtained by EBUS-TBNA in particular yielded sufficient amounts of DNA. Our findings suggest that, even if only small tissue specimens are collected, frozen cytology pellets might be useful for NGS analysis.

### Supplementary Information

**Supplementary Material 1.**

**Supplementary Material 2.**

**Supplementary Material 3.**

## Data Availability

The datasets used and/or analyzed during the current study are available from the corresponding author on reasonable request.

## Abbreviations

NGS: Next-generation sequencing
EBUS-TBNA: Endobronchial ultrasound-guided transbronchial needle aspiration
EGFR: Epidermal growth factor receptor
EML-4: Echinoderm microtubule-associated protein-like 4
ALK: Anaplastic lymphoma kinase
ROS1: ROS proto-oncogene 1
BRAF: V-raf murine sarcoma viral oncogene homolog B1BRAF
MET: Mesenchymal-epithelial transition factor
FFPE: Formalin-fixed paraffin-embedded
LCCP: Lung cancer compact panel
LC-SCRUM-Asia: Lung Cancer Genomic Screening Project for Individualized Medicine in Asia
ECOG: Eastern Cooperative Oncology Group
EBB: Endobronchial biopsy
TBB: Transbronchial biopsy
EBUS-GS-TBB: Endobronchial ultrasound with a guide sheath transbronchial biopsy
NSCLC: Non-squamous non-small cell lung cancer
ODxTT: Oncomine Dx Target Test
KRAS: KRAS proto-oncogene
HER2: Human epidermal growth factor receptor 2
NRAS: Neuroblastoma RAS viral (v-ras) oncogene homolog
HRAS: Harvey rat sarcoma viral oncogene homolog
PIKC3A: Phosphatidylinositol-4,5-bisphosphate 3-kinase catalytic subunit alpha
FGFR: Fibroblast growth factor receptor
RET: Rearranged during transfection
NTRK: Neurotrophic receptor tyrosine kinase
NRG1: Neuregulin-1
GNAS: GNAS complex locus
ROSE: Rapid on-site cytologic evaluation
