# Supplementary material for: Utility of bronchoscopically obtained frozen cytology pellets for next-generation sequencing
Source: BMC Cancer. 2024 Apr 17;24:489. doi: 10.1186/s12885-024-12250-5 (PMC11022476; doi:10.1186/s12885-024-12250-5)
Supplement: Supplementary file 1 — Supplementary Material 1. [file 12885_2024_12250_MOESM1_ESM.docx]

Supplementary Table 1: the number of biopsies in TBB-methods

|  | Frozen Cytology Pellet (n=44) | Frozen Tissue (n=54) |
| --- | --- | --- |
| Procedure | median [range] | median [range] |
| TBB (ultrathin) | 5 | 11 |
| EBUS-GS-TBB (thin) | 9 [6-12] | 5.5 [2-10] |
| EBUS-GS-TBB (thick) | 7 [5-9] | 5.5 [4-11] |
| EBB | 5 [4-8] | 5 [3-5] |
| TBB  under X-ray fluoroscopy | 6 [2-10] | 6 [2-11] |
