# Supplementary material for: Utility of bronchoscopically obtained frozen cytology pellets for next-generation sequencing
Source: BMC Cancer. 2024 Apr 17;24:489. doi: 10.1186/s12885-024-12250-5 (PMC11022476; doi:10.1186/s12885-024-12250-5)
Supplement: Supplementary file 2 — Supplementary Material 2. [file 12885_2024_12250_MOESM2_ESM.pdf]

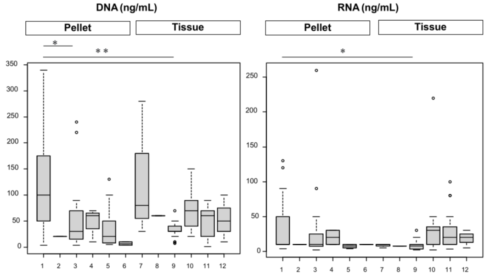

1-EBUS-TBNA, 2-TBB (ultrathin), 3-EBUS-GS-TBB (thin), 4-EBUS-GS-TBB (thick), 5-EBB, 6-TBB under X-ray fluoroscopy, 7-EBUS-TBNA, 8-TBB (ultrathin), 9-EBUS-GS-TBB (thin), 10-EBUS-GS-TBB (thick), 11-EBB, 12-TBB under X-ray fluoroscopy

*$P \leq 0.05$; **$P \leq 0.01$
