# Supplementary material for: Utility of bronchoscopically obtained frozen cytology pellets for next-generation sequencing
Source: BMC Cancer. 2024 Apr 17;24:489. doi: 10.1186/s12885-024-12250-5 (PMC11022476; doi:10.1186/s12885-024-12250-5)
Supplement: Supplementary file 3 — Supplementary Material 3. [file 12885_2024_12250_MOESM3_ESM.docx]

Supplementary Table2: Concordance of NGS analysis by cytology pellets and FFPE

| n=22 |  | NGS analysis by FFPE | |
| --- | --- | --- | --- |
| NGS analysis by frozen cytology pellet |  | success | failure |
| success (n=16) | EBUS-TBNA (n=10) | 9 | 1 |
|  | TBB methods (n=6) | 6 | 0 |
| failure (n=6) | EBUS-TBNA (n=2) | 2 | 0 |
|  | TBB methods (n=4) | 4 | 0 |
